# Supplementary material for: Tissue Engineering as a Promising Treatment for Glottic Insufficiency: A Review on Biomolecules and Cell-Laden Hydrogel
Source: Biomedicines. 2022 Nov 30;10(12):3082. doi: 10.3390/biomedicines10123082 (PMC9775346; doi:10.3390/biomedicines10123082)
Supplement: Supplementary file 1 [file biomedicines-10-03082-s001.zip › biomedicines-1971571-supplementary.pdf]

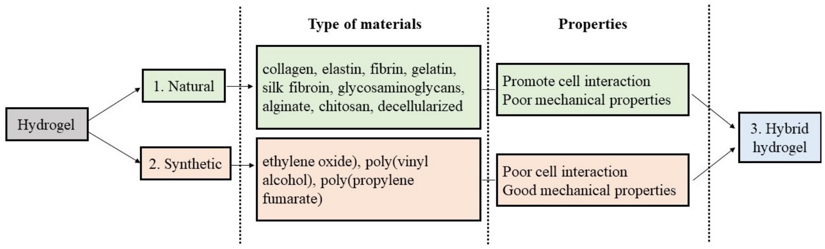

Figure S1: Type of hydrogel

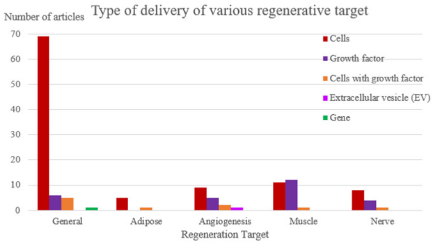

Figure S2: Type of delivery of various regenerative target

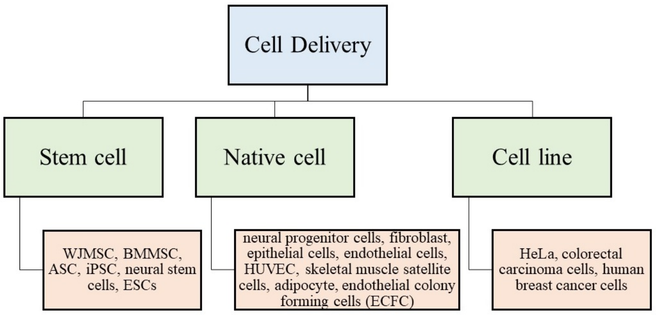

Figure S3: Type of cell delivery via hydrogel

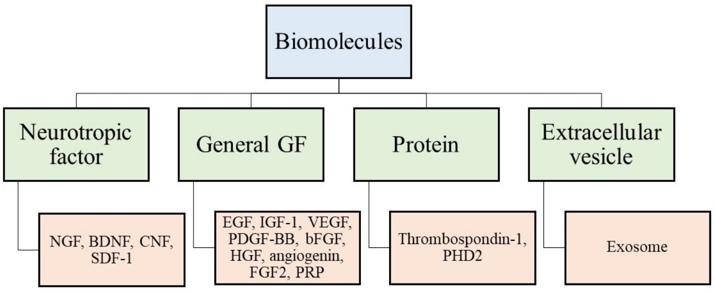

Figure S4: Type of biomolecule delivery via hydrogel.
